# Supplementary material for: End Sequence Analysis Toolkit (ESAT) expands the extractable information from single-cell RNA-seq data
Source: Genome Res. 2016 Oct;26(10):1397–410. doi: 10.1101/gr.207902.116 (PMC5052061; doi:10.1101/gr.207902.116)
Supplement: Supplemental Material [file supp_gr.207902.116_Supp_Materials.tar.gz › ESAT-esat_v0.1/doc/html/functions_o.html]

ESAT: Class Members


ESAT


- Main Page
- Related Pages
- Packages
- Classes
- Files

- Class List
- Class Index
- Class Hierarchy
- Class Members

- All
- Functions
- Variables
- Enumerator

- a
- b
- c
- d
- e
- f
- g
- h
- i
- j
- k
- l
- m
- n
- o
- p
- q
- r
- s
- t
- u
- v
- w
- z


All Classes Namespaces Files Functions Variables Enumerator Pages

Here is a list of all class members with links to the classes they belong to:

### - o -

- offset
  : broad.core.sequence.AbstractFastaParser
- omegaEMIteration()
  : broad.core.siphy.EvolutionaryModel
- or()
  : umms.core.general.Predicates
- orientationForGene()
  : broad.pda.gene.GeneTools
- orientationFromSpliceSites()
  : broad.pda.gene.GeneTools
- OrientedChromosomeTranscriptGraph()
  : umms.core.scripture.OrientedChromosomeTranscriptGraph
- ORIGINAL\_ARRAY\_LEFT
  : broad.pda.seq.rap.CountProbes
- ORIGINAL\_ARRAY\_RIGHT
  : broad.pda.seq.rap.CountProbes
- OUTER\_LENGTH
  : broad.pda.seq.rap.PlotAggregateRegions
- outOrientedDegree()
  : broad.pda.seq.graph.ChromosomeWithBubblesJGraphT
- OUTPUT
  : broad.pda.samtools.FilterPairedReadsByMAQ
  , broad.pda.samtools.PairedEndTDF
  , broad.pda.samtools.TrimFastqReads
  , broad.pda.seq.rap.BuildRatioNullDistribution
  , broad.pda.seq.rap.CalculateCoverageStats
  , broad.pda.seq.rap.CollectAnnotationEnrichments
  , broad.pda.seq.rap.CountProbes
  , broad.pda.seq.rap.CountReads
  , broad.pda.seq.rap.DistanceToBED
  , broad.pda.seq.rap.GCContent
  , broad.pda.seq.rap.PlotAggregateRegions
  , broad.pda.seq.rap.RatioPermutationPeakCaller
  , broad.pda.seq.rap.rna.CountReads
  , broad.pda.seq.rap.ShuffleBAM
  , broad.pda.seq.rap.ShuffleBED
  , broad.pda.seq.rap.SkellamKLPeakCaller
  , broad.pda.seq.rap.SlideAndCalculate
  , broad.pda.seq.rap.SlideAndCount
  , broad.pda.seq.rap.UnmarkDuplicates
- OUTPUT\_1
  : broad.pda.samtools.FilterFastqReads
- OUTPUT\_2
  : broad.pda.samtools.FilterFastqReads
- overlap()
  : broad.pda.gene.GeneTools
- OVERLAP
  : broad.pda.seq.rap.BuildRatioNullDistribution
  , broad.pda.seq.rap.CountProbes
  , broad.pda.seq.rap.PlotAggregateRegions
  , broad.pda.seq.rap.RatioPermutationPeakCaller
  , broad.pda.seq.rap.SkellamKLPeakCaller
  , broad.pda.seq.rap.SlideAndCalculate
  , broad.pda.seq.rap.SlideAndCount
- overlap\_GenomicLevel()
  : broad.pda.annotation.BEDFileParser
- overlapByGenomicRegion()
  : broad.pda.annotation.BEDFileParser
- OverlapFilter()
  : umms.core.annotation.filter.OverlapFilter
- OverlapIterator()
  : broad.core.datastructures.IntervalTree< V >.OverlapIterator
- overlappers()
  : broad.core.datastructures.IntervalTree< V >
- overlappingValueIterator()
  : broad.core.datastructures.IntervalTree< V >
- overlaps()
  : broad.core.annotation.BasicLightweightAnnotation
  , broad.core.multiplealignment.MAFAlignment
  , broad.core.multiplealignment.MultipleAlignment
  , broad.core.sequence.SequenceRegion
  , broad.pda.gene.GeneWithIsoforms
  , broad.pda.seq.graph.Path
  , umms.core.annotation.AbstractAnnotation
  , umms.core.annotation.Annotation
  , umms.core.annotation.BasicAnnotation
  , umms.core.annotation.CompoundInterval
  , umms.core.annotation.Gene
  , umms.core.annotation.SingleInterval
- overlaps2()
  : umms.core.annotation.Gene
- overlapsAtAll()
  : broad.pda.datastructures.Alignments
- overlapsByGenomicRegion()
  : broad.pda.gene.GeneWithIsoforms
- overlapsCollection()
  : broad.pda.datastructures.Alignments
- overlapsExon()
  : broad.pda.gene.GeneWithIsoforms
  , umms.core.annotation.Gene
- overlapsGene()
  : umms.core.annotation.Gene
- overlapsGeneInAnyOrientation()
  : umms.core.annotation.Gene
- overlapsLeft()
  : broad.pda.seq.graph.Path
- overlapsRight()
  : broad.pda.seq.graph.Path
- overlapsStranded()
  : broad.core.sequence.SequenceRegion
  , umms.core.annotation.AbstractAnnotation
  , umms.core.annotation.Annotation
  , umms.core.annotation.Gene


---

Generated on Fri Aug 15 2014 15:11:16 for ESAT by  

 1.8.7
